# Atypical presentation of celiac disease with ascites and hypoalbuminemia mimicking malignancy

**DOI:** 10.1093/omcr/omag014

**Published:** 2026-03-23

**Authors:** Salma El Aouadi, Basma Dghoughi, Soukaina Bahha, Ouaim Taibi, Hajar Oubella, Ola Messaoud, Omar El Aoufir, Laila Jroundi, Zaynab Iraqi Houssaini

**Affiliations:** Emergency Radiology Department, Ibn Sina University Hospital, Mohammed V University, Souissi District, Rabat 10000, Rabat-Salé-Kénitra Region, Morocco; Emergency Radiology Department, Ibn Sina University Hospital, Mohammed V University, Souissi District, Rabat 10000, Rabat-Salé-Kénitra Region, Morocco; Emergency Radiology Department, Ibn Sina University Hospital, Mohammed V University, Souissi District, Rabat 10000, Rabat-Salé-Kénitra Region, Morocco; Emergency Radiology Department, Ibn Sina University Hospital, Mohammed V University, Souissi District, Rabat 10000, Rabat-Salé-Kénitra Region, Morocco; Gastroenterology Department, Ibn Sina University Hospital, Mohammed V University, Souissi District, Rabat 10000, Rabat-Salé-Kénitra Region, Morocco; Emergency Radiology Department, Ibn Sina University Hospital, Mohammed V University, Souissi District, Rabat 10000, Rabat-Salé-Kénitra Region, Morocco; Emergency Radiology Department, Ibn Sina University Hospital, Mohammed V University, Souissi District, Rabat 10000, Rabat-Salé-Kénitra Region, Morocco; Emergency Radiology Department, Ibn Sina University Hospital, Mohammed V University, Souissi District, Rabat 10000, Rabat-Salé-Kénitra Region, Morocco; Emergency Radiology Department, Ibn Sina University Hospital, Mohammed V University, Souissi District, Rabat 10000, Rabat-Salé-Kénitra Region, Morocco

**Keywords:** celiac disease, peritoneal carcinomatosis, hypoalbuminemia, ascites

## Abstract

Celiac disease is an autoimmune disorder of the small intestine that usually presents with gastrointestinal symptoms; however, atypical extraintestinal manifestations can occur, making diagnosis challenging. We report a unique case of celiac disease presenting with generalized edema, ascites, and elevated CA-125 levels, closely simulating an underlying malignancy. Imaging revealed diffuse ascites and mesenteric lymphadenopathy without the typical intestinal features of celiac disease. The diagnosis was ultimately confirmed by serology and small bowel biopsy. This case underscores the diagnostic challenge of atypical presentations and highlights the importance of early recognition. Celiac disease should be considered in the differential diagnosis of unexplained ascites with elevated CA-125 to avoid unnecessary oncologic workup and delayed treatment.

## Introduction

Celiac disease, also known as gluten-sensitive enteropathy or celiac sprue, is an autoimmune disorder characterized by inflammation and villous atrophy of the small intestine [1]. It affects about 1%–2% of the global population and is more frequently diagnosed in women [1]. While it typically presents with gastrointestinal symptoms such as diarrhea, abdominal pain, and malabsorption, a wide range of atypical extraintestinal manifestations—including anemia, osteoporosis, and hepatic dysfunction—has been described [2]. Severe hypoalbuminemia with generalized edema and ascites is exceptionally rare and may mimic a malignant process [3]. Such presentations pose significant diagnostic challenges, and this case adds to the limited reports of celiac disease mimicking malignancy.

## Case report

A 44-year-old woman with insulin-dependent diabetes mellitus was admitted for the evaluation of unexplained edema and ascites evolving over three months. Her symptoms began with progressive bilateral leg edema, followed by abdominal distension. She denied gastrointestinal bleeding, chronic diarrhea, vomiting, or weight loss. On examination, she had tense ascites and bilateral lower limb edema extending to the thighs, without hepatosplenomegaly, lymphadenopathy, or cardiovascular abnormalities.

The initial laboratory workup showed normal complete blood count, liver function tests and renal profile including serum creatinine, 24-h urinary protein excretion, and complement levels. Cardiac assessment using electrocardiogram and transthoracic echocardiography was also normal. Screening for immune deficiencies, including QuantiFERON testing and viral serologies (HBsAg, anti-HCV, and HIV), was negative. However, profound hypoalbuminemia was detected, with serum albumin level of 18 g/l. Tumor markers were also assessed, revealing elevated CA-125 (Cancer Antigen) levels at 95 U/ml, while CA 19–9 and carcinoembryonic antigen (CEA) were within normal ranges.

**Figure 1:**
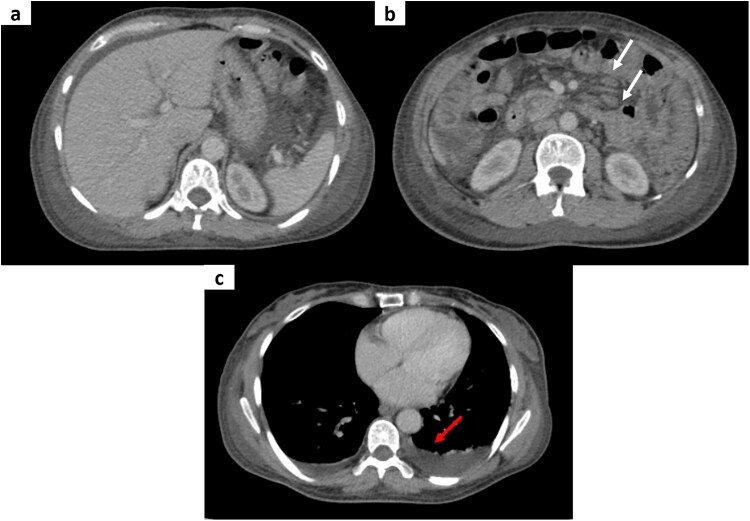
Axial contrast-enhanced CT images of the abdomen and pelvis in the portal phase (a–c) demonstrating extensive ascites with associated stranding and infiltration of the peritoneal fat, along with edematous changes in the subcutaneous tissues. Multiple homogeneous mesenteric lymph nodes are visible (white arrows). Bilateral pleural effusions are also present (red arrow).

Abdominal ultrasound revealed large-volume peritoneal effusion. Contrast-enhanced computed tomography (CT) of the chest, abdomen, and pelvis (Fig. 1) confirmed extensive diffuse ascites involving all peritoneal compartments, associated with stranding and infiltration of the peritoneal fat and edematous changes in the subcutaneous tissues. Multiple mildly enlarged but homogeneous mesenteric lymph nodes were observed, without discrete masses or focal lesions. Bilateral pleural effusions were also observed. Diagnostic paracentesis revealed an exudative ascitic fluid with high protein content and a low serum–ascites albumin gradient (SAAG).

**Figure 2:**
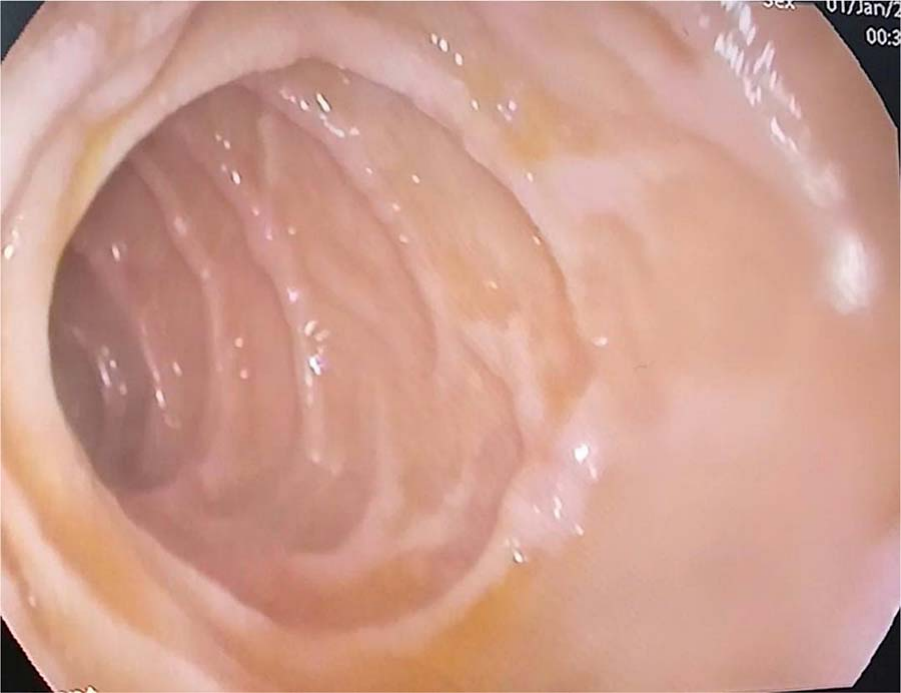
Endoscopic image of the duodenum showing normal mucosa with preserved duodenal folds.

Given these findings along with elevated CA-125 levels, secondary peritoneal carcinomatosis was suspected. To search for a primary tumor, pelvic magnetic resonance imaging (MRI) was performed but did not reveal any suspicious masses or peritoneal nodules. The upper gastrointestinal endoscopy was macroscopically normal (Fig. 2), and colonoscopy did not reveal any abnormalities. However, random biopsies were also performed.

**Figure 3:**
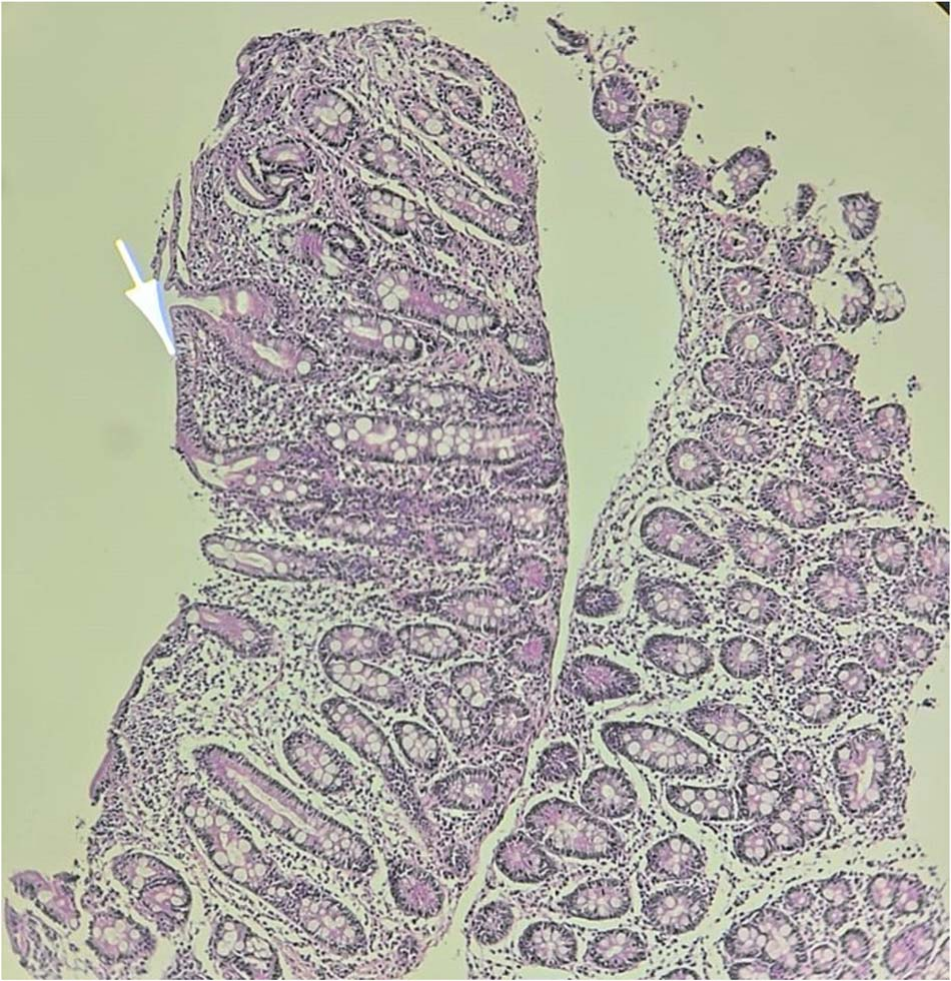
Histopathological image of the duodenal biopsy at low magnification showing villous atrophy and crypt hyperplasia.

Histological examination of duodenal biopsies demonstrated villous atrophy with crypt hyperplasia, consistent with celiac disease (Fig. 3). To confirm this unexpected diagnosis, serological testing was performed, which revealed that anti-tissue transglutaminase IgA antibodies were strongly positive at levels greater than 10 times the upper normal limit. Anti-endomysial antibodies and HLA typing were not performed.

**Figure 4:**
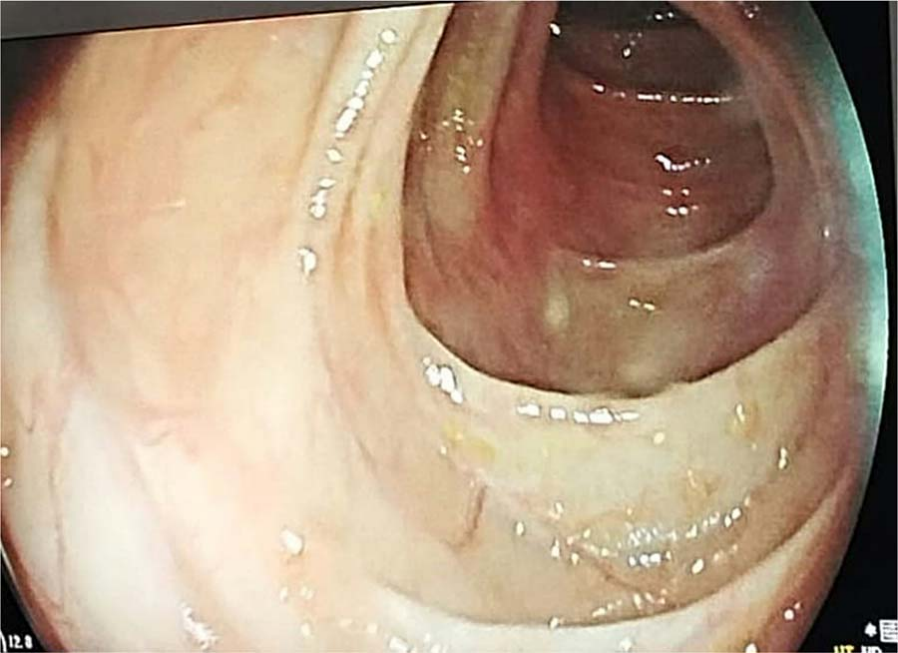
Normal colonoscopy showing no mucosal abnormalities.

The patient initially received albumin infusions, resulting in rapid improvement in her general condition. Subsequently, a strict gluten-free diet was administered. At follow-up, her clinical status improved significantly, with complete regression of ascites and peripheral edema, disappearance of pleural effusion, and normalization of serum albumin from 18 g/l to 37 g/l. Follow-up colonoscopy was normal (Fig. 4). Overall, her evolution was favorable under dietary management, with recovery of general well-being and significant improvement in nutritional parameters.

## Discussion

Celiac disease is a common inflammatory condition of the small intestine resulting from an immune-mediated reaction to gluten in genetically predisposed individuals [1]. Diagnosis relies on serological testing—particularly anti-tissue transglutaminase IgA—and is confirmed by duodenal biopsy showing increased intraepithelial lymphocytes, crypt hyperplasia, and villous atrophy [1].

Clinical presentation can be highly variable. Typical presentations involve gastrointestinal complaints such as diarrhea, abdominal pain, and malabsorption, while atypical forms manifest with extraintestinal features [2, 4]. Severe hypoalbuminemia with generalized edema and ascites is exceptionally rare and has been described only in a few reports (Table 1) [3, 5, 6]. These atypical features significantly contribute to the diagnostic complexity.

**Table 1:** Summary of published cases reporting hypoalbuminemia and edema as atypical presentation of celiac disease.

Author	Age/Sex	Main Clinical Presentation	Notes
Ernoić et al. [3]	84-year-old female	Severe hypoalbuminemia, generalized edema, ascites	Atypical presentation; no classic GI symptoms
Meena et al. [5]	56-year-old male	Generalized edema, abdominal distension	Extraintestinal onset; initial absence of GI symptoms
Barakat et al. [6]	4-year-old male	Acute generalized edema, severe hypoalbuminemia; no GI symptoms	Pediatric atypical case
Present case	Adult female	Generalized edema, severe hypoalbuminemia, massive ascites, elevated CA-125	Mimicked peritoneal carcinomatosis; no typical intestinal features

Hypoalbuminemia is often associated with malignancy, through malnutrition, systemic inflammation, or protein-losing enteropathy [3]. In addition, tumor markers such as CA-125 may be elevated in non-malignant conditions involving severe inflammation or hypoalbuminemia, further complicating the picture [3]. Consequently, the combination of ascites, hypoalbuminemia, and elevated CA-125 levels strongly suggests an underlying malignant process.

Ascites in celiac disease is primarily explained by protein-losing enteropathy, which reduces plasma oncotic pressure and favors third spacing of fluids [1]. Additional mechanisms such as increased intestinal permeability, mucosal inflammation, and capillary leak may further contribute to fluid accumulation despite normal hepatic function [1, 2].

Cross-sectional imaging plays a key role in evaluating suspected celiac disease because manifestations vary according to disease stage and may involve intestinal and extraintestinal sites [7]. Typical intestinal abnormalities include jejunal fold loss, luminal dilation, increased fluid content, delayed transit, and occasionally transient intussusception or intramural fat deposition [7]. Extraintestinal manifestations may include mesenteric lymphadenopathy, splenic atrophy, or—rarely—ascites [1, 7].

In our patient, imaging showed diffuse ascites and homogeneous mesenteric lymphadenopathy, findings that initially raised concern for peritoneal carcinomatosis given the elevated CA-125. However, the absence of typical malignant features—such as peritoneal nodularity, or irregular thickening—made this diagnosis less likely and maintained the possibility of an atypical presentation of celiac disease.

The main treatment for celiac disease is a strict gluten-free diet, which alleviates symptoms, reduces antibodies, and prevents complications [8]. Patients require monitoring for nutritional deficiency, dietary adherence, and new symptoms. Emerging immunologic therapies are under investigation, and multidisciplinary support is important because of their lifelong dietary and psychosocial impact [1, 9].

This case illustrates that ascites with marked hypoalbuminemia can occur in celiac disease, that elevated CA-125 is not specific for malignancy, and that discordant imaging and laboratory findings should prompt consideration of benign etiologies, including atypical celiac disease.

## Conclusion

This case demonstrates that celiac disease rarely presents with ascites, hypoalbuminemia, and elevated tumor markers, mimicking malignancy. Awareness of such atypical presentations and careful imaging evaluation are essential for accurate diagnosis, allowing timely initiation of a gluten-free diet and clinical improvement.
